# Trends in the level and composition of supplemental benefits in Medicare Advantage

**DOI:** 10.1093/haschl/qxad019

**Published:** 2023-06-20

**Authors:** Grace McCormack, Erin Trish

**Affiliations:** Schaeffer Center for Health Policy and Economics, University of Southern California, Los Angeles, CA 90089, United States; Schaeffer Center for Health Policy and Economics, Pharmaceutical and Health Economics, Mann School of Pharmacy, University of Southern California, Los Angeles, CA 90089, United States

**Keywords:** Medicare Advantage, supplemental benefits, insurance

## Abstract

Medicare Advantage (MA) plans that bid below benchmarks (or bidding targets) receive a portion of that difference as rebates, which they then must return to beneficiaries through supplemental benefits or reduced premiums or cost-sharing. Using Centers for Medicare & Medicaid Services data, we evaluate the growth in rebates and concomitant changes in supplemental benefit composition among health maintenance organizations (HMOs) and local preferred provider organizations (PPOs) from 2011 through 2022. Average rebates grew considerably, particularly after 2015 and among PPOs. Alongside this rebate growth, the share of enrollees in plans offering dental, vision, and hearing benefits also increased, with nearly universal coverage of these benefits among both HMOs and PPOs by 2022. Medicare Advantage plans also increasingly reduced beneficiary Part D premium obligations, while increasing beneficiary financial exposure in the form of higher Part D deductibles, medical out-of-pocket maximums, and cost-sharing for inpatient stays. These findings are particularly relevant as policymakers debate the merits of various reforms to MA payment policy.

## Introduction

There has been considerable policy attention focused on Medicare Advantage (MA), particularly on plan payments. The Medicare Payment Advisory Commission (MedPAC) estimates that, in 2022, these payments averaged up to 4% more than what it would have cost the Medicare program were the same beneficiaries instead enrolled in traditional Medicare, despite the fact that MA plans’ bids suggest they can provide coverage for approximately 12% less than traditional Medicare costs, on average.^1^

**Figure 1.**
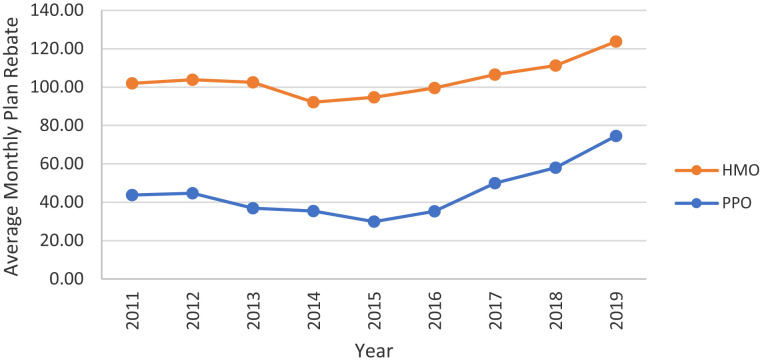
Average monthly Medicare Advantage (MA) rebate, by plan type, 2011–2019. Averages are enrollment weighted using data from the Centers for Medicare & Medicaid Services (CMS) MA landscape files. Results are presented in nominal dollars. Source: Authors’ analysis of the CMS plan payment files. Abbreviations: HMO, health maintenance organization; PPO, preferred provider organization.

However, when MA plans bid below benchmarks (or bidding targets), they receive a portion of that difference in the form of rebates, which they then must return to beneficiaries through supplemental benefits or reduced premiums or cost-sharing.^2^ Over the past decade, plans have garnered increasingly large rebates (Figure 1) due to declining bids relative to benchmarks, suggesting that increasing dollars are available to fund these various extra benefits. While recent analyses have evaluated the prevalence of MA plans offering certain supplemental benefits—such as vision, dental, and hearing coverage^3^—and the extent to which this varies across MA organizations,^4^ there has been limited analysis of how the magnitude and composition of such benefits have evolved over time. But understanding these trends is useful to inform MA payment policy debates and evaluate the potential impact of reforms on beneficiaries, particularly in light of the considerable growth in both MA rebates and MA enrollment over the last decade.^5^

## Data and methods

We used the Centers for Medicare & Medicaid Services (CMS) Plan Benefits Package files from 2011 to 2022 as our primary data source on MA supplemental benefits. We linked these with data on MA premiums from the CMS landscape files (over the same time period) and rebate payments from the CMS plan payment files, which are only currently available through 2019. Additionally, to enrollment-weight our analyses, we linked these data with enrollment information from the CMS MA landscape files. We exclude Special Needs Plans and Employer Group Health Plans as well as enrollees residing in Puerto Rico and other territories. Additionally, we restrict our analyses to health maintenance organizations (HMOs) and local preferred provider organizations (PPOs).

We group our main analyses by financial and in-kind supplemental benefits. For analyses of in-kind benefits, we focus primarily on those related to vision (eye exams, eyewear), dental (preventative, comprehensive), and hearing (hearing exams, hearing aids). For our analyses of financial benefits, we focus on beneficiary Part D premium, Part D deductible, medical out-of-pocket maximum (MOOP), and inpatient co-pays. Most MA enrollment is in plans that offer prescription drug coverage,^3^ and plans can use their rebate dollars to reduce the Part D premium and deductible that the beneficiary would otherwise pay. Unlike traditional Medicare, MA plans have a statutory MOOP for medical services that was set at $6700 for most of the study period, and increased to $7550 in 2020, but plans can use their rebate dollars to offer limits below this threshold. Most MA plans waive the inpatient hospital deductible and instead typically charge a daily copayment.^3^ We present financial results in nominal dollars (ie, not adjusted for inflation).

Additionally, in supplemental analyses, we evaluate the provision of special supplemental benefits for the chronically ill (SSBCI) in 2022. Since 2020, MA plans have had additional leeway to offer these SSBCI benefits that are not primarily health related.

## Results

From 2011 to 2019 enrollment-weighted average MA rebates per member per month increased on net for both HMOs (from $101.97 in 2011 to $123.77 in 2019) and PPOs (from $43.75 to $74.58) (Figure 1). However, rebates modestly shrank in the first half of the decade before rebounding with sharp growth post-2015. While average rebates for HMOs were consistently higher than for PPOs, the difference has diminished in recent years due to the relatively faster growth among PPOs.

**Figure 2.**
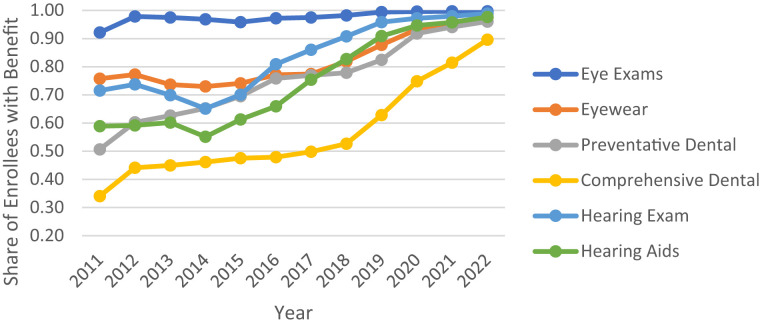
Share of Medicare Advantage (MA) enrollment in plans offering specified in-kind benefits: HMOs, 2011–2022. Source: Authors’ analysis of the Centers for Medicare & Medicaid Services (CMS) plan benefits package files and the CMS MA landscape files. Abbreviation: HMO, health maintenance organization.

**Figure 3.**
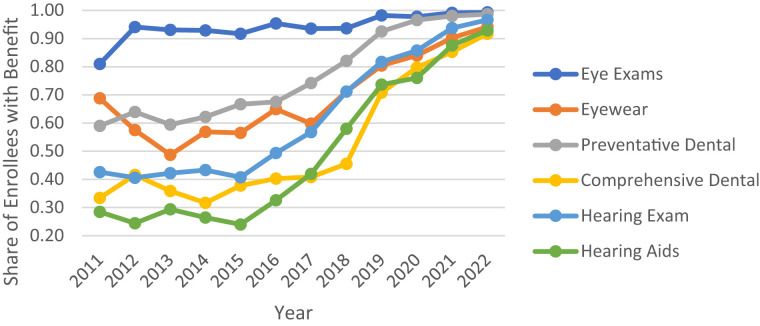
Share of Medicare Advantage (MA) enrollment in plans offering specified in-kind benefits: PPOs, 2011–2022. Source: Authors’ analysis of the Centers for Medicare & Medicaid Services (CMS) plan benefits package files and the CMS MA landscape files. Abbreviation: PPO, preferred provider organization.

Alongside this rebate growth, the share of enrollees in plans offering dental, vision, and hearing benefits also increased from 2011 through 2022 (Figures 2 and 3). While coverage for eye exams has been nearly universal for HMO and PPO enrollees over this time period, coverage for eyewear, dental, and hearing benefits increased considerably, particularly among PPOs. By 2022, even comprehensive dental coverage—historically the least common of the major supplemental benefits—was covered for 90% of HMO enrollees and 92% of PPO enrollees (Appendix Exhibit 1).^6^ High coverage rates of these in-kind benefits were pervasive across most counties in 2022, even among those that had relatively lower average rebates or low levels of MA market competition (Appendix Exhibits 2, 3, and 5).^6^

**Figure 4.**
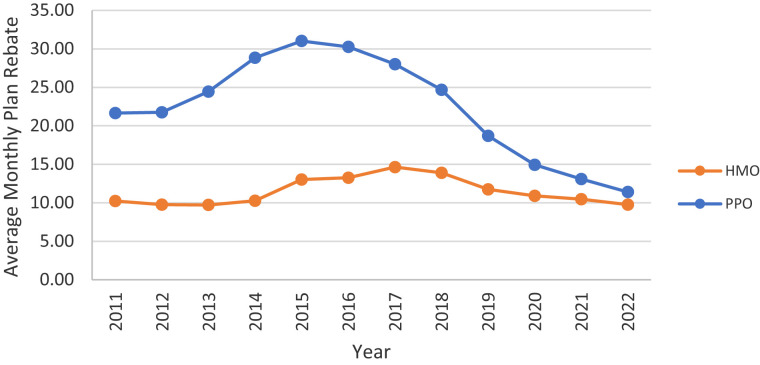
Average monthly beneficiary-paid Part D premium, by plan type: 2011–2022. Beneficiary-paid Part D premiums are net of any buy-down using rebate dollars. Averages are enrollment weighted using data from the Centers for Medicare & Medicaid Services (CMS) Medicare Advantage (MA) landscape files. Source: Authors’ analysis of the CMS plan benefits package files. Results are presented in nominal dollars. Abbreviations: HMO, health maintenance organization; PPO, preferred provider organization.

**Figure 5.**
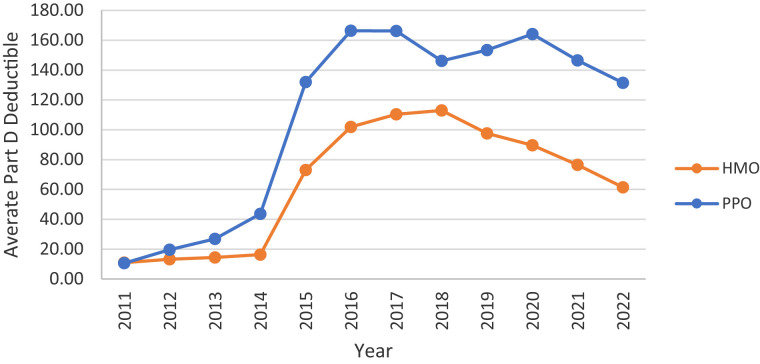
Average annual Part D deductible, by plan type: 2011–2022. Averages are enrollment weighted using data from the Centers for Medicare & Medicaid Services (CMS) Medicare Advantage (MA) landscape files. Source: Authors’ analysis of the CMS plan benefits package files. Results are presented in nominal dollars. Abbreviations: HMO, health maintenance organization; PPO, preferred provider organization.

**Figure 6.**
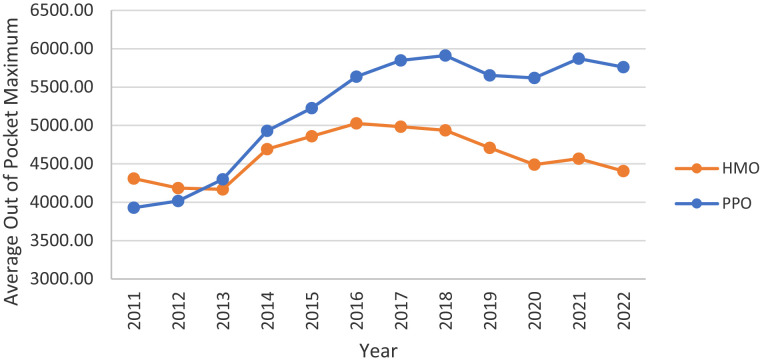
Average annual out-of-pocket maximum, by plan type, 2011–2022. Averages are enrollment weighted using data from the Centers for Medicare & Medicaid Services (CMS) Medicare Advantage (MA) landscape files. Source: Authors’ analysis of the CMS plan benefits package files. Results are presented in nominal dollars. Abbreviations: HMO, health maintenance organization; PPO, preferred provider organization.

**Figure 7.**
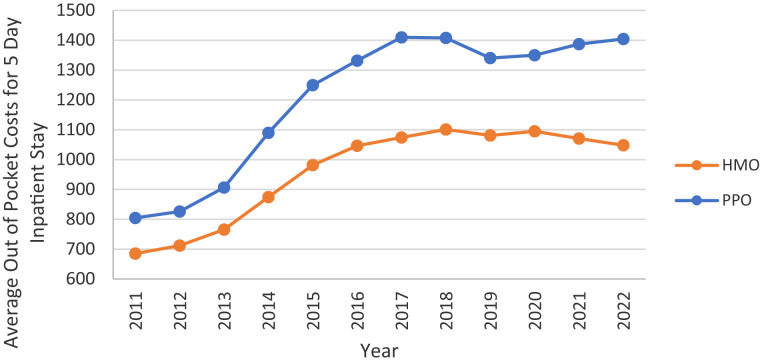
Average out-of-pocket costs for a 5-day inpatient stay, by plan type: 2011–2022. Averages are enrollment weighted using data from the Centers for Medicare & Medicaid Services (CMS) Medicare Advantage (MA) landscape files. Source: Authors’ analysis of the CMS plan benefits package files. Results are presented in nominal dollars. Abbreviations: HMO, health maintenance organization; PPO, preferred provider organization.

In contrast, the provision of financial benefits varies considerably across plan types (Figures 4–7). While average Part D premiums (net of any buy-down with rebate dollars) were relatively low for HMOs throughout the study period, they decreased considerably among PPOs, from a high of $31.02 per month in 2015 to $11.40 in 2022 (Figure 4). In contrast, average Part D deductibles increased among both HMOs and PPOs, particularly after 2014, with higher average annual deductibles among PPOs ($131.39) than HMOs ($61.48) in 2022 (Figure 5).

While many plans offer MOOPs below the required threshold, the average has increased among both HMOs and PPOs, albeit more so among PPOs. Despite PPOs actually having a lower average MOOP ($3928.70) than HMOs ($4307.75) in 2011, by 2022, the average was considerably higher among PPOs ($5762.09) than HMOs ($4405.48). Similarly, the average out-of-pocket co-pay for a 5-day inpatient stay increased for both HMO and PPO enrollees over this time period (Figure 7).

While counties with higher average rebates tend to offer more generous financial benefits, particularly for Part D premium and deductible reductions, there is considerable variation and counties tend not to be universally generous across all financial benefit types studied (Appendix Exhibit 4).^6^ Further, there is no consistent cross-sectional relationship between financial benefits and MA market competition (Appendix Exhibit 6).

In addition to these standard supplemental benefits, a small share of 2022 MA enrollment was in a plan that offered at least 1 SSBCI benefit (Appendix Exhibit 4).^6^ Overall, coverage of these SSBCI benefits is low among the plans in our sample, although it is higher among HMOs than PPOs (15% of HMOs covered any SSBCI benefit in 2022 vs 10% of PPOs) (Appendix Exhibit 7), and notably higher among Elevance (formerly Anthem) plans than other organizations (Appendix Exhibit 8).^6^ In 2022, the most common SSBCI offered was the provision of food, produce, and meals (Appendix Exhibit 4).^6^

## Discussion

This analysis of trends in MA rebates and supplemental benefits offers several key insights. First, overall rebates grew, on net, from 2011 to 2019, and MedPAC analyses indicate that this trend has continued.^7^ This suggests that MA plans are spending fewer dollars relative to traditional Medicare on medical benefits covered by Parts A and B and are, instead, funneling increasing dollars into supplemental benefits and beneficiary cost reductions that are not covered by traditional Medicare. Second, while HMOs today receive 40% higher rebates than PPOs, the gap has narrowed over time, suggesting that—while starting from a higher baseline—PPO bids are declining (relative to benchmarks) more quickly than HMO bids. Put another way, the difference between these 2 plan types—at least along the efficiency demonstrated through their bids—appears to be narrowing over time.

Third, the overall level and composition of supplemental benefit provision has shifted considerably over the last decade. Coverage of dental, vision, and hearing benefits is now nearly universal in MA, reflecting substantial increases, particularly among PPOs. Medicare Advantage plans also increasingly used their rebate dollars to reduce beneficiary Part D premium obligations, in contrast with rising beneficiary financial exposure in the form of higher Part D deductibles,^8^ medical out-of-pocket maximums, and cost-sharing for inpatient stays. These findings are consistent with previous research that MA plans have historically focused their rebate dollars toward plan characteristics that are particularly salient to consumers at the time of their enrollment decision.^9,10^ Indeed, revealed preferences indicate that beneficiaries value these guaranteed cost savings; in 2022, 69% of enrollees in MA plans that offered Part D coverage chose a plan that fully paid their Part D premium.

Finally, uptake of plans offering newly permitted SSBCI benefits was relatively low among the plans in our sample. This finding is consistent with other analyses that have found SSBCI benefit offerings to be particularly concentrated among Special Needs Plans,^4,11^ which are excluded from our sample, although our findings indicate that offering these benefits as part of standard MA plans is a strategy unique to Elevance.

These results are particularly salient as policymakers debate MA payment reform, particularly in light of the divide between plan bids and payments resulting from the current payment system. Our findings suggest that rebates have grown considerably, and that they are increasingly being used to fund a number of in-kind supplemental benefits and Part D premium reductions. While beneficiaries likely value these benefits—and their increasing generosity may have been a driver of the considerable growth in MA enrollment—they have added to divide between the relative benefits offered by MA and traditional Medicare. Indeed, while recent policy efforts to add vision, dental, and hearing benefits to the traditional Medicare package have stalled due to their projected high costs to Medicare, these same benefits have become near universal in MA. However, despite increasing rebates, MA plans have become less generous in terms of important financial protection benefits—out-of-pocket spending maximums, inpatient cost-sharing, and Part D deductibles. The composition of MA enrollees has changed over this time and thus the effective generosity—for example, as a share of spending—may not have diminished at the same rate as the levels themselves. Nonetheless, taken together, our findings signal a shift toward benefits that are likely more salient at the point of enrollment but perhaps less generous in terms of insurance value for medical benefits covered under Parts A and B.

The welfare impact of this shift in benefit provision depends upon multiple factors, including how these various types of benefits impact enrollee health, satisfaction, and health care costs. Past work suggests that Medicare beneficiaries value in-kind supplemental benefits when making enrollment decisions.^11^ However, additional work is needed to better quantify the potential enrollment effects of policy changes that could affect MA benefits in today's market. Such an understanding would be beneficial for modeling the impact of potential reforms to the MA program, particularly because recent evidence suggests that a reduction in benchmarks for MA plans would likely result in a modest reduction in the provision of both in-kind and financial benefits.^12^ Beyond their effects on enrollment, there is mixed evidence on how well these in-kind supplemental benefits facilitate utilization or overall health.^13–15^ Similarly, while financial characteristics may be less salient to consumers, increased cost-sharing could have a negative impact on enrollees, not only by increasing out of pocket costs but also by reducing utilization of high-value health care services.^16^

Our findings are limited in that we did not evaluate all supplemental benefits offered by MA plans nor do we have data on their utilization. Further, among the supplemental benefits we did study, we did not assess the generosity of supplemental benefits conditional on coverage (eg, among plans that covered dental benefits, we did not assess the generosity of cost-sharing or extent of services covered). Similarly, we did not assess plans on other dimensions—such as the breadth of their provider networks—which may also have been changing over this time period.

Nonetheless, it is clear that MA rebate dollars have increased considerably over the last decade, alongside plan generosity on multiple dimensions, particularly among PPOs. As policymakers debate the merits of MA payment reform, future work is needed to better understand the extent to which enrollees are benefiting from these extra benefits—particularly relative to their costs—and the extent to which changes to MA payment policy might impact plan quality, generosity, enrollment, and federal spending.^17^

## Supplementary Material

qxad019_Supplementary_Data
