# Supplementary material for: Trends in the level and composition of supplemental benefits in Medicare Advantage
Source: Health Aff Sch. 2023 Jun 20;1(1):qxad019. doi: 10.1093/haschl/qxad019 (PMC10986227; doi:10.1093/haschl/qxad019)
Supplement: qxad019_Supplementary_Data [file qxad019_Supplementary_Data.zip › appendix_figures.pdf]

(1) 2022 Plan Characteristics and Benefit Provision by Plan Type

	Total Enrollment	Eye Exams	Eye Wear	Preventative Dental	Comprehensive Dental	Hearing Exam	Hearing Aids
HMO	11758876	1.00	0.97	0.96	0.90	0.99	0.98
Local_PPO	6188616	0.99	0.94	0.99	0.92	0.97	0.93

	Average Part D Premium	$0 Part D Premium	Average Drug Deductible	$0 Drug Deductible	Max Out of Pocket	Avg OOP Costs 5 Day Inpatient Stay	Any SSBCI
HMO	9.76	0.73	61.48	0.76	4405.48	1048.53	0.15
Local_PPO	11.40	0.65	131.39	0.42	5762.09	1403.79	0.10

This table shows average provision of various supplemental benefits that can be funded by rebate dollars. The sample of plans is restricted to non-SNP, non-EGWP HMO and Local PPO plans offered in the 50 United States and the District of Columbia 2022. The first column shows total enrollment in either plan type in 2022. The subsequent columns show the enrollee-weighted average of plan provision of various benefits. The first row shows the share of enrollees in plans that offer eye exams, eyewear (e.g., glasses), preventative dental care, comprehensive dental care, hearing exams, and hearing aids. Similarly, the second row shows enrollee-weighted averages of various plan characteristics. In order, the columns show the average Part D premium for plans offering Part D benefits, the share of enrollees in plans that offer a $0 premium Part D plan, the average Part D drug deductible, the share of enrollees in plans that offer a $0 Part D drug deductible, the average out of pocket maximum for medical coverage, the average out of pocket cost for a five day inpatient stay, and the share of enrollees in plans offering any Special Supplemental Benefits for the Chronically Ill (SSBCI). All financial outcomes are in nominal dollars.

(2) County Averages
(Weighted by Enrollees)

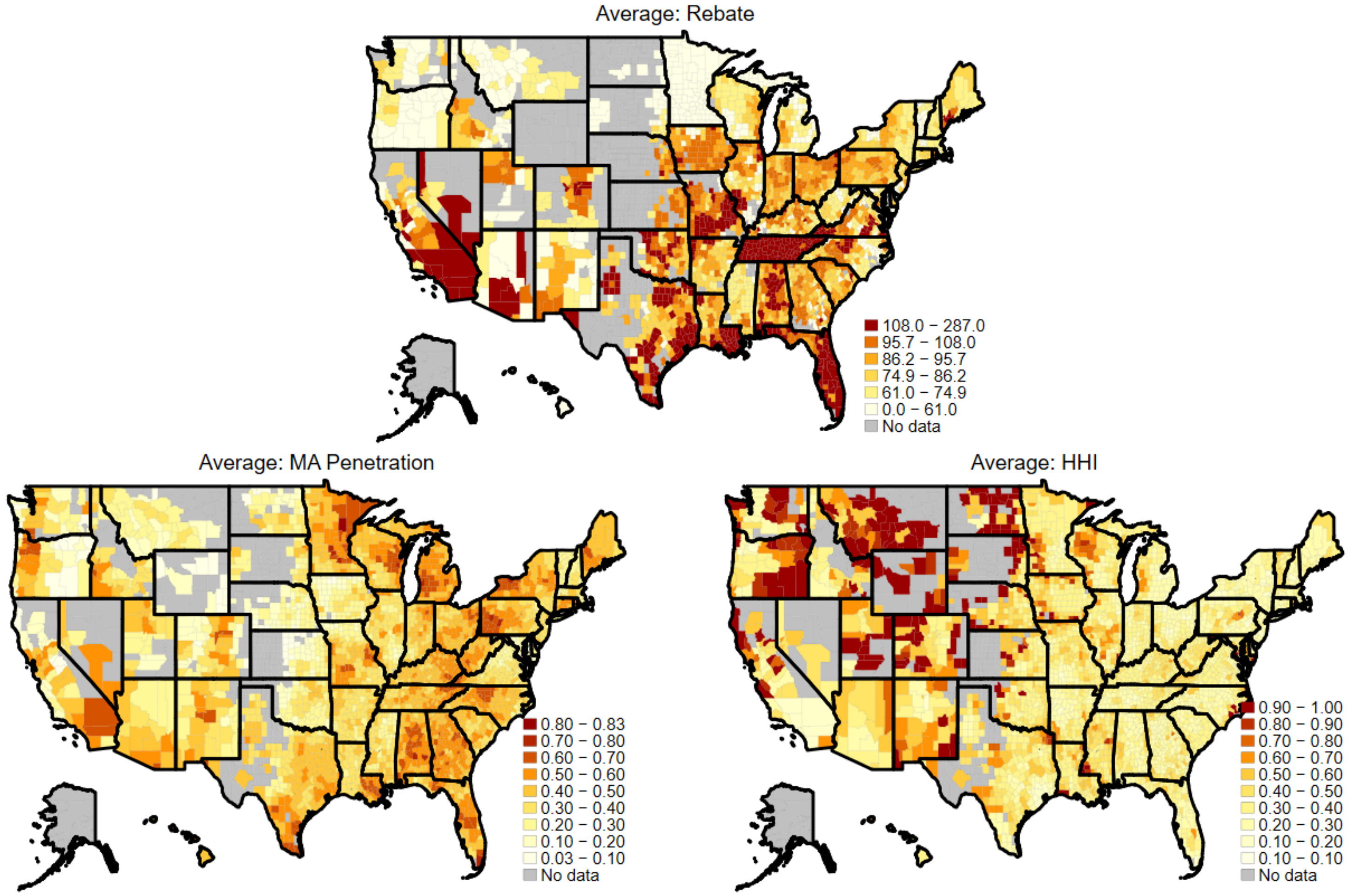

This figure shows various county level averages in the United States. The first panel shows the enrollee-weighted average rebates in each county in 2019, the latest year that rebate data is available. The bottom two panels show the average MA penetration (i.e. share of Medicare population in MA) and MA HHI, respectively in 2022. HHI is calculated using the market share for carrier i in a given county c, S_{ic}. For a county with carriers $i = 1, ...I$, we calculate HHI $= \sum_{i=1}^{I} S_{ic}$. For all panels in the figure, the sample of plans is restricted to non-SNP, non-EGWP HMO and Local PPO plans offered in the 50 United States and the District of Columbia.

(3) 2022 County Averages of In-Kind Benefits
(Weighted by Enrollees)

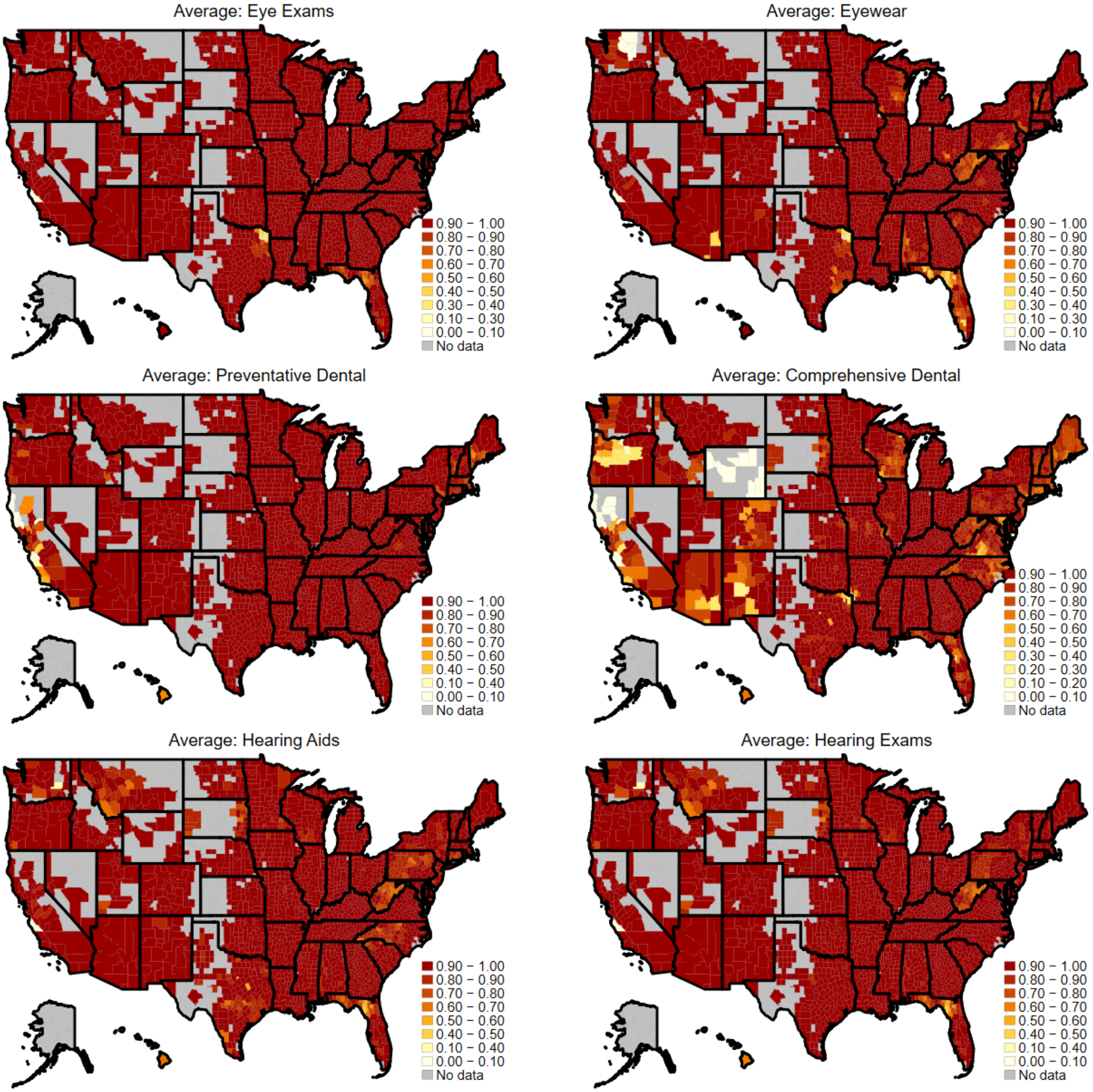

This figure shows the enrollee-weighted average of benefit provision in each county in the United States in 2022. The sample of plans is restricted to non-SNP, non-EGWP HMO and Local PPO plans offered in the 50 United States and the District of Columbia 2022.

(4) 2022 County Averages of Financial Benefits
(Weighted by Enrollees)

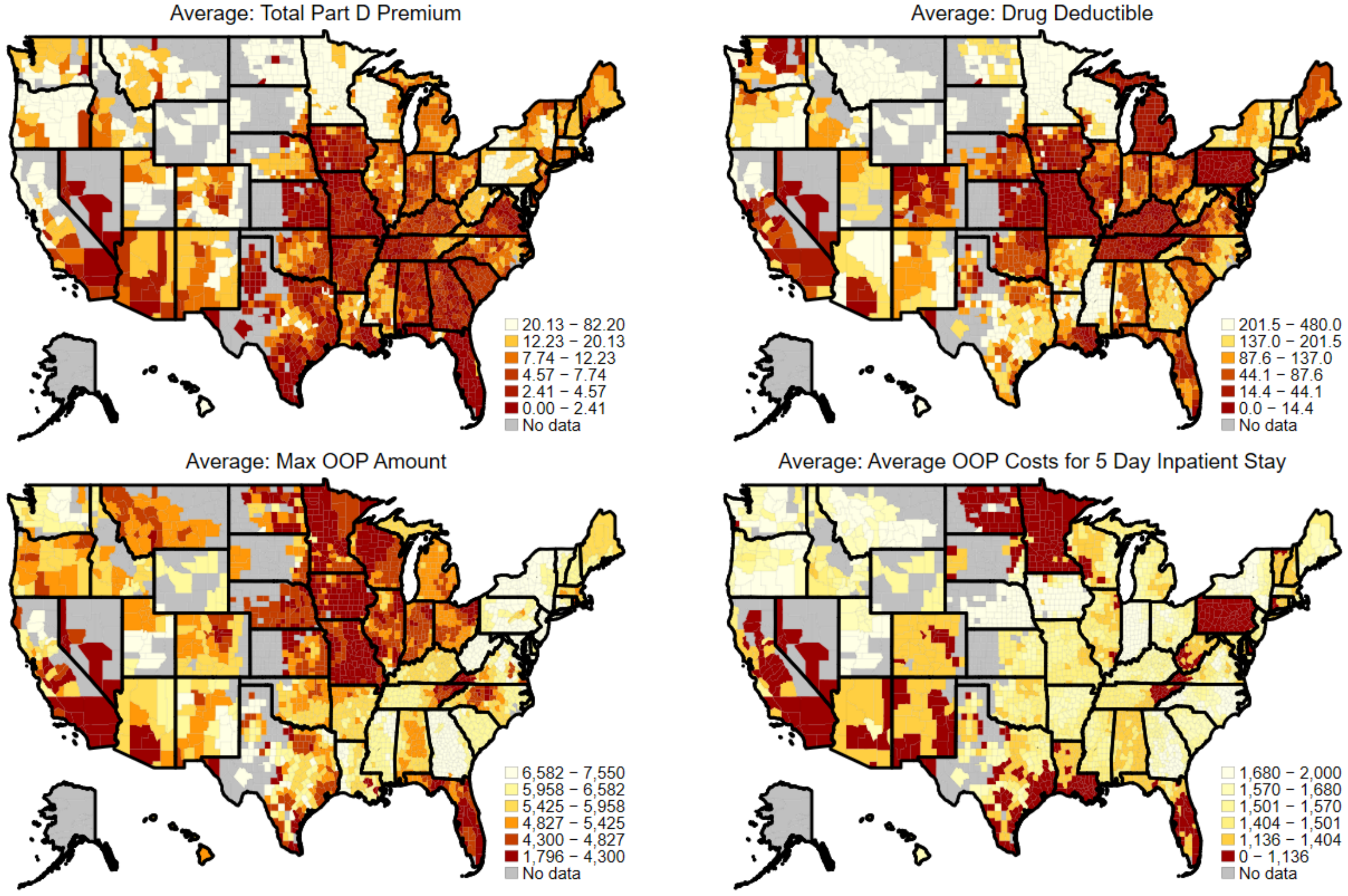

This figure shows the enrollee-weighted average of various plan financial characteristics in each county in the United States in 2022. All financial outcomes are in nominal dollars. The sample of plans is restricted to non-SNP, non-EGWP HMO and Local PPO plans offered in the 50 United States and the District of Columbia 2022.

(5) 2022 In-Kind Benefits vs. HHI
(Weighted by Enrollees)

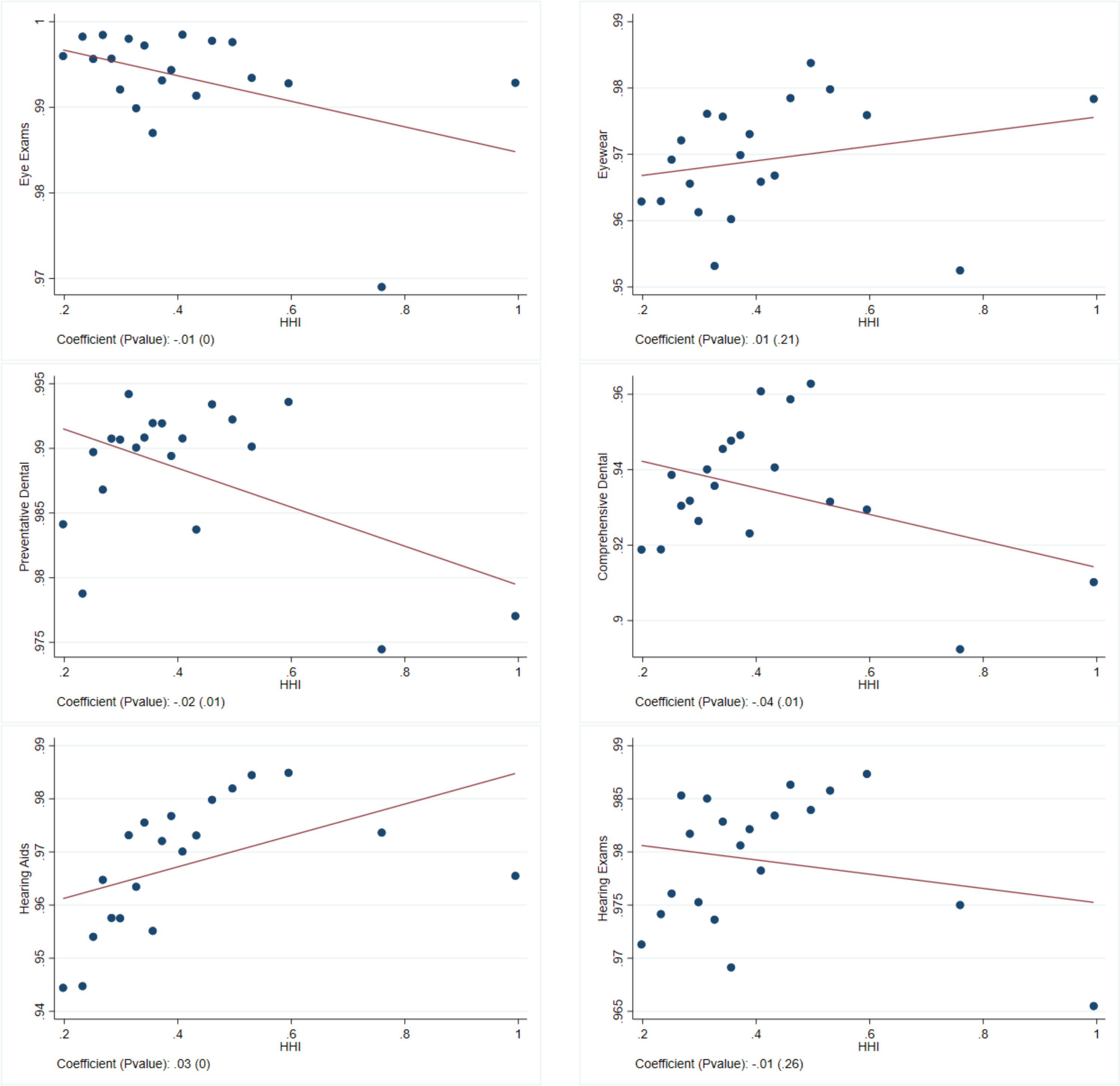

This figure shows a binscatter between the enrollee-weighted average of benefit provision in each county with respect to the HHI in that same county in the United States in 2022. HHI is calculated using the market share for carrier i in a given county c, S_{ic}. For a county with carriers $i = 1, \ldots I$, we calculate HHI $= \sum_{i=1}^{I} S_{ic}$. The coefficient and p-value of the linear relationship between these two statistics are displayed below each sub-figure. The sample of plans is restricted to non-SNP, non-EGWP HMO and Local PPO plans offered in the 50 United States and the District of Columbia 2022.

(6) 2022 Financial Benefits vs. HHI
(Weighted by Enrollees)

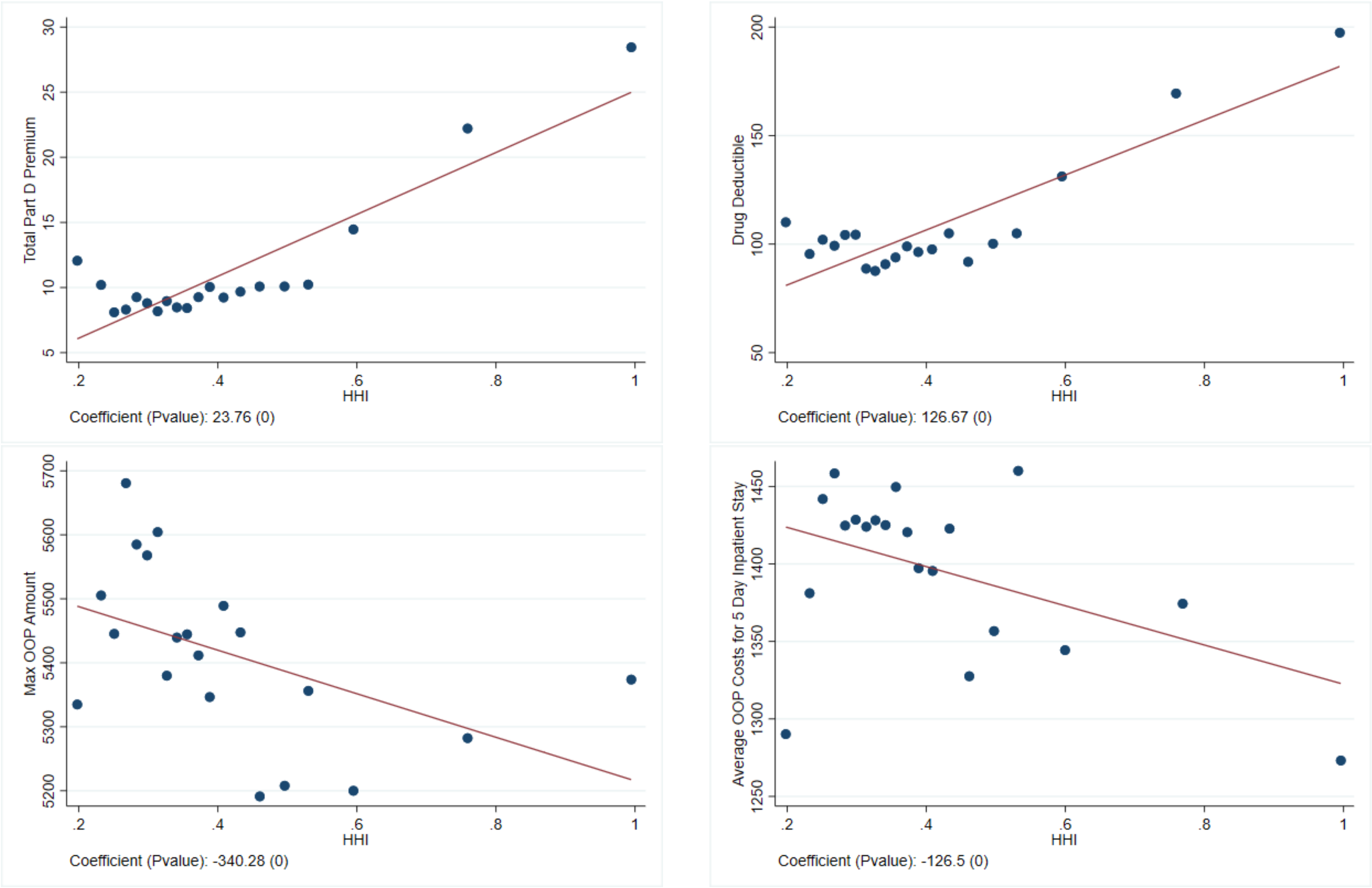

This figure shows a binscatter between the enrollee-weighted average of benefit provision in each county with respect to the HHI in that same county in the United States in 2022. HHI is calculated using the market share for carrier i in a given county c, S_{ic}. For a county with carriers $i = 1, ...I$, we calculate HHI $= \sum_{i=1}^{I} S_{ic}$. All financial outcomes are in nominal dollars. The coefficient and p-value of the linear relationship between these two statistics are displayed below each sub-figure. The sample of plans is restricted to non-SNP, non-EGWP HMO and Local PPO plans offered in the 50 United States and the District of Columbia 2022.

(7) 2022 SSBCI Provision By Plan Type
(Enrollee Weighted)

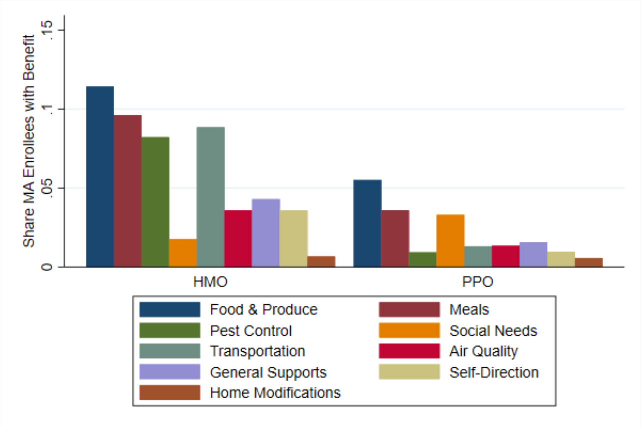

This figure shows the enrollee-weighted average of plan provision of various Special Supplemental Benefits for the Chronically Ill (SSBCI) in 2022. The sample of plans is restricted to non-SNP, non-EGWP HMO and Local PPO plans offered in the 50 United States and the District of Columbia 2022.

(8) 2022 SSBCI Provision by Insurer
(Enrollee Weighted)

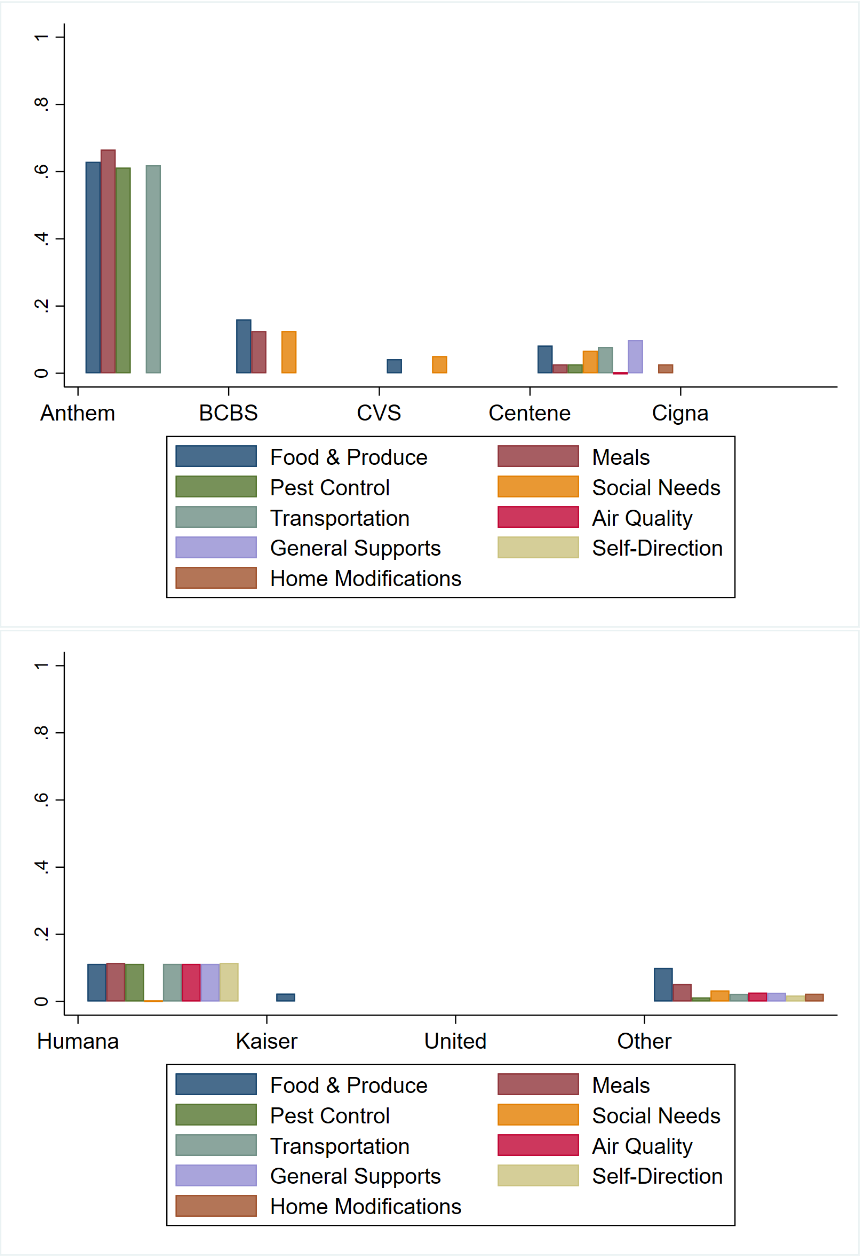

This figure shows the enrollee-weighted average of plan provision of various Special Supplemental Benefits for the Chronically Ill (SSBCI) in 2022 among the largest insurers. The sample of plans is restricted to non-SNP, non-EGWP HMO and Local PPO plans offered in the 50 United States and the District of Columbia in 2022.
